# Missed, Not Missing: Phylogenomic Evidence for the Existence of Avian FoxP3

**DOI:** 10.1371/journal.pone.0150988

**Published:** 2016-03-03

**Authors:** Michael P. Denyer, Dammy Y. Pinheiro, Oliver A. Garden, Adrian J. Shepherd

**Affiliations:** 1 Department of Clinical Sciences and Services, The Royal Veterinary College, London, United Kingdom; 2 Institute of Structural and Molecular Biology and Department of Biological Sciences, Birkbeck, University of London, London, United Kingdom; University of Catania, ITALY

## Abstract

The Forkhead box transcription factor FoxP3 is pivotal to the development and function of regulatory T cells (Tregs), which make a major contribution to peripheral tolerance. FoxP3 is believed to perform a regulatory role in all the vertebrate species in which it has been detected. The prevailing view is that FoxP3 is absent in birds and that avian Tregs rely on alternative developmental and suppressive pathways. Prompted by the automated annotation of *foxp3* in the ground tit (*Parus humilis*) genome, we have questioned this assumption. Our analysis of all available avian genomes has revealed that the *foxp3* locus is missing, incomplete or of poor quality in the relevant genomic assemblies for nearly all avian species. Nevertheless, in two species, the peregrine falcon (*Falco peregrinus)* and the saker falcon *(F*. *cherrug*), there is compelling evidence for the existence of exons showing synteny with *foxp3* in the ground tit. A broader phylogenomic analysis has shown that FoxP3 sequences from these three species are similar to crocodilian sequences, the closest living relatives of birds. In both birds and crocodilians, we have also identified a highly proline-enriched region at the N terminus of FoxP3, a region previously identified only in mammals.

## Introduction

Peripheral tolerance mechanisms are an essential part of the adaptive immune system, controlling inflammation and preventing inappropriate immune responses to autoantigens, fetal alloantigens and gastrointestinal microflora [1,2]. The Forkhead box transcription factor FoxP3 plays a key role in the development and function of regulatory T cells (Tregs), which make a major contribution to peripheral tolerance [3].

FoxP3 belongs to the Fox family of transcription factors, which share an 80 to 100 amino acid DNA-binding domain known as the forkhead box [4]. The forkhead (FRK) domain is the only part of the protein sequence that can be aligned across all members of the Fox family and is thus a critical determinant in phylogenetic studies [5]. The closest relatives to FoxP3 are FoxP1, FoxP2 and FoxP4. All four proteins share a zinc finger (ZF), leucine zipper (LZ) and FRK domain. The distinguishing features of FoxP3 include the C terminal position of the FRK domain, compared with a central position in other FoxP family members [6], and, in mammals, a disordered proline-rich N-terminal (ProR) region that contrasts with the glutamine-rich N-terminal region of other FoxP molecules [6,7].

FoxP3 has been identified in a diversity of vertebrate species, including numerous mammals [8–15], bony fish (Osteichthyes) [16–23] and amphibians [24]. In all these studies, the molecule is thought to serve a regulatory role. However, despite the recent release of a dataset of 48 consistently annotated avian genomes, of which prior versions of the zebra finch (*Taeniopygia guttata*) and chicken (*Gallus gallus*) genomes featured in a detailed phylogenomic investigation of FoxP3 [7], the prevailing view in the literature has always been that FoxP3 is absent in birds and that their Tregs rely on alternative developmental and suppressive pathways [25,26].

Publication of the genome of the ground tit (*Parus humilis*) in January 2013 was ground-breaking in revealing a number of adaptations of this species to extreme altitude, including rapidly evolving hypoxia response genes [27,28]. The high genome coverage and quality of the sequencing data in this species prompted us to re-visit the issue of FoxP3 loss in birds.

We present evidence supporting the automated annotation of FoxP3 in the ground tit, including its presence in two additional avian genomes. We also provide a plausible explanation for the failure to identify FoxP3 in dozens of other avian genomes and offer preliminary data suggesting that the ProR region is not only present in extant archosaurs (birds and crocodilians) but is also particularly proline-enriched in these species. These findings prompt a sea-change in our view of the evolution of avian immune tolerance and Treg mechanisms.

## Materials and Methods

### Sequence data

All vertebrate canonical-form FoxP1 to FoxP4 protein sequences orthologous to the corresponding murine Foxp sequences were retrieved from Ensembl [29] in November 2014. Sequences from additional vertebrate species were retrieved from the NCBI reference sequence database [30]; where multiple isoforms were present, the longest isoform was retrieved.

To understand syntenic regions surrounding the putative *foxp3* gene in the ground tit, the DNA region containing *foxp3* together with its flanking genes was downloaded from the NCBI, as were the corresponding mRNA and protein sequences, yielding the following set of putative genes: *foxp3* (ID: 102110738); the nearest upstream gene on the same strand, *naa10* (an N-alpha-acetyltransferase 10, NatA catalytic subunit gene; ID: 102108725); the nearest downstream gene on the same strand, *cacna1f* (calcium channel, voltage-dependent, L type, alpha 1F subunit; ID: 102111116); the nearest gene at the 5’ end of *foxp3* on the opposing DNA strand, *ppp1r3f* (a protein phosphatase 1, regulatory subunit 3F gene; ID: 106628755); and the nearest gene at the 3’ end of *foxp3* on the opposing DNA strand, *ccdc22* (coiled-coil domain containing 22; ID: 102110932). In the American crow (*Corvus brachyrhynchos*), no *naa10* or *naa10*-like gene was identified, so the nearest upstream gene selected was *hsd17b10* (hydroxysteroid 17-beta-dehydrogenase 10; ID: 103617130). Orthologous sequences of these genes in all available avian species were retrieved from NCBI using BLASTN [31], together with assembly statistics for whole avian genomes. BLASTP searches were also performed on CrocBase [32,33] using FoxP3 of the American alligator (*Alligator mississippiensis*) to identify orthologous *foxp3* loci in other crocodilians.

TBLASTN [34] searches on the NCBI dbEST [35] were performed for chicken, wild duck (*Anas platyrhynchos*) and zebra finch using the ground tit FoxP3 protein. (EST sequences for the ground tit or peregrine [*Falco peregrinus*] and saker [*F*. *cherrug*] falcons are not available.) For comparison, ESTs for pig (*Sus scrofa)* were interrogated using the porcine FoxP3 sequence from Ensembl (ENSSSCP00000030259).

PCR primers used in previous experimental work that sought to identify FoxP3 in the chicken were acquired from the authors [36]. A BLASTN search [34] of the primers was then undertaken against ground tit *foxp3*.

### Sequence analysis

When at least one *foxp3*-flanking gene and at least one fragment of DNA involving the putative *foxp3* locus was retrieved for a given avian genome, GeneWise [37]–a tool for comparing a genomic DNA sequence with a protein sequence—was used to detect exons showing partial or complete matches to FoxP3 in the ground tit. Exon boundaries were manually curated with the ground tit sequence. The same procedure was undertaken for the genomes of crocodilians, using the X2 isoform of FoxP3 of the American alligator (*A*. *mississippiensis*) as the reference. FoxP1 to FoxP4 protein sequences were aligned using MUSCLE [38] and viewed in Jalview [39]. All NCBI BLAST searches were carried out using versions 2.2.30+ to 2.2.32+ and either the nr/nt, nr, SRA or est database.

## Results and Discussion

### Putative *foxp3* loci are missing or incomplete in most avian genomes

Automated annotations of FoxP3 were found only in the turkey (*Meleagris gallopavo*; Ensembl) and ground tit (*P*. *humilis*; NCBI annotation release 100). BLASTP analysis against the NCBI nr database suggested that the annotation of the ground tit gene was correct, with top matches to mammalian and reptilian FoxP3; in contrast, that of the turkey gene was erroneous, with top matches to avian FoxP4. Given the large number of published avian genomes, we considered that a single automated gene annotation was weak evidence for the presence of FoxP3 in birds. For example, an alternative explanation might be genomic contamination [40]. Moreover, in the subsequent ground tit NCBI annotation release 101, the automated annotation of the ground tit was removed.

Why, then, do we still support the presence of FoxP3 in the ground tit? Gene prediction software such as Softberry FGENESH [41] still predicts that *foxp3* is present in the inter-genic region between *ppp1r3f* and *ccdc22* (S1 File). The presence of *cacna1f* and *ccdc22* lends credence to the ground tit prediction, since the same genes are found in close proximity to *foxp3* in mammals, reptiles and amphibians (Table 1); the turkey gene incorrectly annotated as *foxp3* belongs to a scaffold with no flanking genes. Importantly, while vertebrate *foxp1-4* are descended from the same ancestral locus [42], paralogues to *cacna1f* and *ccdc22* are not found in the gene regions of *foxp1*, *foxp2* and *foxp4* supporting the evolutionary preservation of the *foxp3* genomic region.

**Table 1:** The genomic conservation of *foxp3* with its downstream flanking genes, *cacna1f* and *ccdc22*.

Class	No. of genomes	*cacna1f*	*ccdc22*	*foxp3*	*foxp3* annotated *cacna1f* not annotated	*foxp3* annotated *ccdc22* not annotated	*foxp3* not annotate *cacna1f* annotated	*foxp3* not annotate *ccdc22* annotated
Mammals^a^	105	87	86	88	1^b^	2^b^^,^^c^	0	0
Amphibians	2	1	1	1	0	0	0	0
Reptiles^d^	13	5	6	4	0	0	1^d^	2^d^^,^^e^

Summary data showing the consistent association between *foxp3* and its two downstream flanking genes, *ccdc22* (or *ccdc22*-like) on the opposing strand and *cacna1f* (or *cacna1f*-like) on the same strand. Summary data was compiled from all NCBI scaffold and chromosome assembled genomes (S1 Table).

^a^Five NCBI genomes had multiple scaffolds in this region: the Weddell seal (*Leptonychotes weddellii*) had an annotation of *cacna1f* with a downstream gap before gene *ppp1r3f*, which in mammals is typically found downstream of *foxp3*, and had *foxp3* and *ccdc22* annotated on a separate scaffold (and terminating upstream of *ccdc22*); there were two cases where *ccdc22* and *cacna1f* are separated; there was one case where *ccdc22* and *foxp3* are separated; and one case where there are partial *ccdc22* annotations on two scaffolds. Approximately a quarter of mammalian genomes also had another gene or pseudogene (e.g. heat shock protein family B (small) member 1 pseudogene 2 in humans) annotated between *ccdc22* and *cacna1f*.

^b^Three missing gene annotations (one *cacna1f* and two *ccdc22*) are attributed to incomplete assemblies in the *foxp3* gene region.

^c^In the gray short-tailed opossum (*Monodelphis domestica*) NCBI genome *ccdc22* is not annotated, but it is annotated in Ensembl.

^d^The western painted turtle (*Chrysemys picta bellii*) was the only identified reptilian genome without the conserved location of both flanking genes, as *cacna1f* and *ccdc22* were both annotated on the same scaffold, but remote from each other. *foxp3* was not annotated. Both alligators also had an inversion of *ccdc22*, but this did not alter the conserved location of this gene with respect to *foxp3* and *cacna1f*.

^e^The garter snake (*Thamnophis sirtalis*) genome was missing *foxp3* and *cacna1f* annotations. However, downstream of *ccdc22*, there is an annotation of *cacna1d*-like with numerous masked repeat regions, hence a possible mis-annotation for *cacna1f*. Upstream the scaffold terminates after *ccdc22* but before the predicted *foxp3* locus.

**Fig 1:**
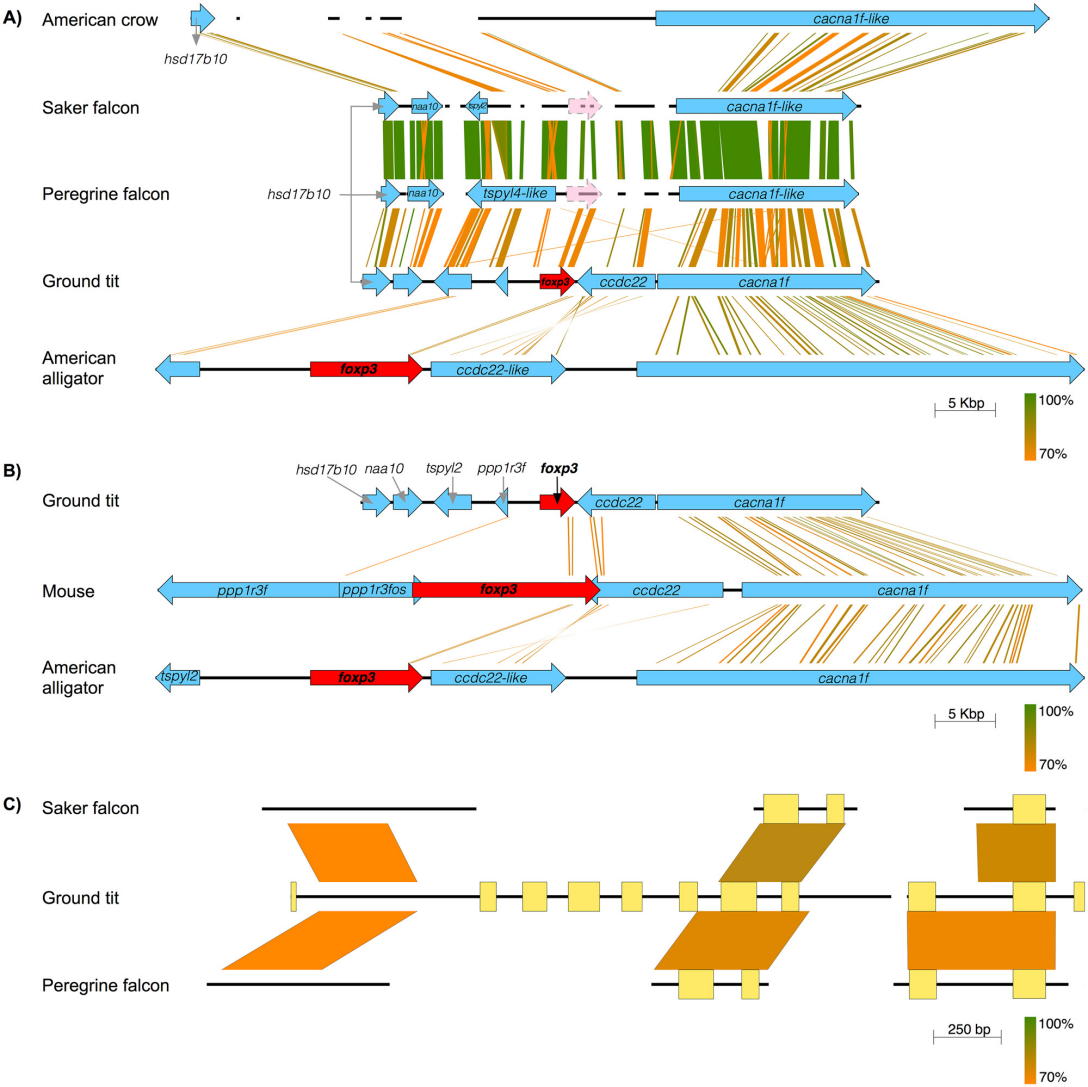
The genomic context of avian *foxp3*. (A) The gene neighbourhood of *foxp3 in* a number of archosaurs showing the significant masked repeats of the American crow between *hsd17b10* and *cacna1f* (gaps in the line indicate intergenic repeat regions), the approximate location of the *foxp3* locus in the saker and peregrine falcon (marked by a pink arrow with a dashed line), and the low nucleotide sequence identity of *foxp3* between the American alligator and the ground tit. In the displayed region the overall masked repeats are as follows: 54.7% in the American crow, 28.0% in the saker falcon, 31.4% in the peregrine falcon, 6.8% in the ground tit and 0.02% in the American alligator. (B) The *foxp3* gene neighbourhood of the mouse, American alligator and ground tit, showing the gene variation upstream of *foxp3* in mammals, birds and crocodilians and the low nucleotide sequence identity of murine *foxp3* versus the American alligator and ground tit. (C) A comparison of the approximate *foxp3* loci in both falcons with the ground tit *foxp3 locus*, showing masked repeats (gaps in the line) and predicted exons (rectangles). All subfigures were produced using Easyfig [47] with BLASTN identity comparisons indicated by the scales on each subfigure.

A syntenic analysis of 60 scaffold or chromosome assembled avian genomes revealed only 21 with an annotated *cacna1f* (or *cacna1f*-like). Of these 21 genes, 11 belonged to single-gene scaffolds and 16 consisted of partial mRNA. Only eight of the genomes had an annotated *naa10* (or *naa10*-like), one of which was a partial gene belonging to a single-gene scaffold. A total of four avian genomes with a single scaffold spanning the putative *foxp3* locus were thus available for interrogation, three of which contained both *cacna1f* (or *cacna1f*-like) and *naa10* (or *naa10*-like) (Fig 1A). The fourth genome, belonging to the American crow (*C*. *brachyrhynchos*), possessed *hsd17b10* (not *naa10*-like) as its nearest annotated downstream gene. Our failure to identify *foxp3* exons between *hsd17b10* and *cacna1f* was attributed to the presence of a large gap of 23,713 bp within the 36,546 bp inter-genic region. However, in the unmasked regions of *foxp3*, introns of the American crow had high nucleotide identity to the other three avian genomes containing both *cacna1f* (or *cacna1f*-like) and *naa10* (or *naa10*-like). These three remaining genomes belonged to the ground tit, peregrine falcon (*F*. *peregrinus*) and saker falcon (*F*. *cherrug*). Of note, all three species had exceptionally high quality genomes, combining a small numbers of scaffolds (<7500), high scaffold N50 values (>3.9 mBP) and uncommonly high genome coverage (>95x) compared to other avian species, as summarised in Table 2. Fifty-four of the 61 available avian genomes, including all three birds with identifiable FoxP3, were sequenced with Ilumina Hiseq, which may be prone to significant errors in certain regions of the genome [43]. Moreover, genome assemblies are often incomplete and error-prone in general [44–46], and the FoxP3 region of the genome presumably presents particular challenges in birds and indeed a number of reptiles.

**Table 2:** Avian genome quality and its relationship to the annotation and assembly of the *foxp3* gene neighbourhood.

	Zero annotated scaffolds	Single gene scaffold(s) only	One or more multi-gene scaffold(s)
No. of genomes	31	15	14
Average coverage	46x	59x	92x
Average scaffold N50	931,132	4,481,081	7,011,777
Average no. of scaffolds	67,526	47,461	47,280

The table shows the association between the quality of avian genomes and the assembly and annotation of the avian *foxp3* gene neighbourhood (*hsd17b10*, *naa10*, *tspyl2*, *ppp1r3f*, *ccdc22* and *cacna1f*). The better the genome quality in the *foxp3* region the easier it is to annotate genes and assemble multi-gene scaffolds (in the absence of a reference sequence). Summary data was compiled from all NCBI scaffold and chromosome assembled genomes (S2 Table).

A recent study argues that high GC content and lengthy G/C-rich regions are key reason why genes in avian genomes have been erroneously reported as missing, and identifies the ground tit genome as being the most complete avian genome in terms of G/C-rich regions [48]. Given that the ground tit region containing the putative *foxp3* gene (i.e. between the end of *naa10* 3’ and the start of *cacna1f* 5’) has 66% GC content, this provides a plausible explanation for the absence of an assembled sequence spanning this region in most avian genomes.

We sought to probe further the failure to detect FoxP3 in key avian species. No matches to ground tit FoxP3 were found in a TBLASTN search of the NCBI dbEST for chicken (~600,000 ESTs), wild duck (~92,000 ESTs) and zebra finch (~3,500 ESTs). However, of note, we were also unable to find porcine FoxP3 within a corresponding set of nearly 1,700,000 porcine ESTs, attributed to the biased coverage of EST data [49]. In parallel with these studies, we investigated a set of PCR primers for FoxP3 derived from zebrafish (*Danio rerio*), pig (*Sus scrofa*), cow (*Bos taurus*) and mouse (*Mus musculus*) that had failed to identify FoxP3 in a previous experimental study of chicken [36]. Twenty-eight out of 29 of these primers had only partial nucleotide sequence matches to the ground tit, with a maximum of 72% sequence identity. The single matching primer (TTGTGCAGGCTCAGGTTG) was a perfect match to the NLSLH amino acid sequence of the FRK domain of the ground tit and two falcon species, but was not accompanied by a complementary forward strand primer. Furthermore, we observed remarkably low nucleotide sequence identity between ground tit and non-avian *foxp3* (Fig 1A and 1B), suggesting that primers designed on the basis of the latter would be unlikely to match the chicken sequence. In addition, primer failure rates as high as 17% are not uncommon [50], and amplification is known to be challenging when the sequence is G/C-rich [48].

**Fig 2:**
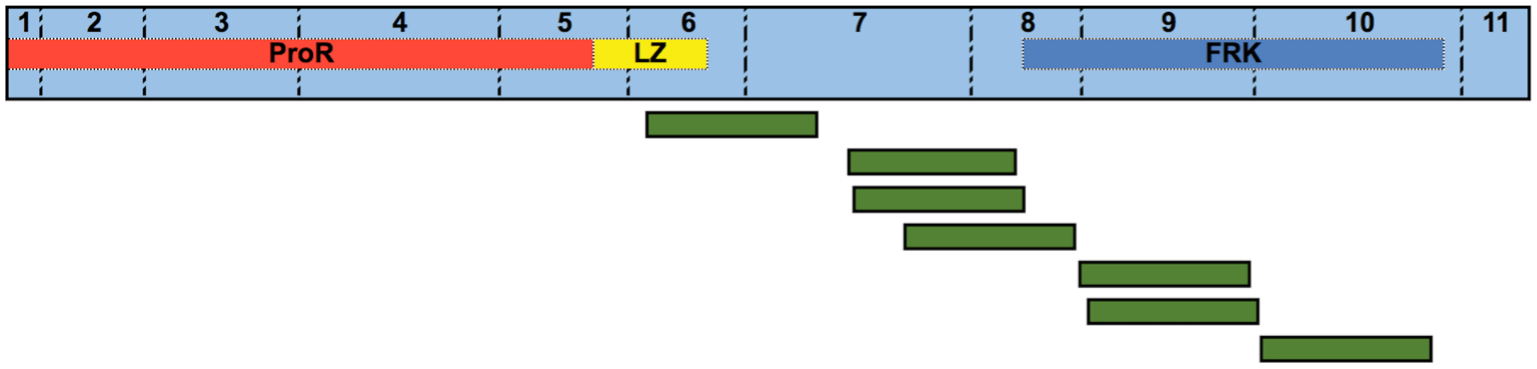
Ground tit *foxp3* transcriptome coverage. The 11 numbered exons of the putative ground tit *foxp3* sequence annotated with the sequence regions that encode ground tit FoxP3 functional regions. A collection of reads with 100% identity (shown in green) were found with a BLASTN search of ground tit RNA-Seq data from the muscle in the SRA (SRX246872; S2 File).

We subsequently identified RNA-Seq data for the ground tit in the Sequence Read Archive (SRA) [51] database and used BLASTN to search within this data using the putative ground tit coding sequence for FoxP3 (S1 File). There were a number of reads with 100% sequence matches spanning the second half of the LZ to the end of the FRK domain (excluding the last four amino acids), with coverage of 144 out of 152 amino acids (Fig 2). Only one of these reads that mapped to the FRK domain was a match to any annotated avian gene, with 96% coverage and 90% identity to ground finch (*Geospiza fortis*) *foxp4*—appreciably less than the 100% coverage and identity of its match to *foxp3*. (Such cross matches to other FRK domains are unsurprising given the high degree of similarity between the FRK domains of *foxp1 to 4*.) Thus, this RNA-Seq dataset provides the strongest evidence to date for avian FoxP3 expression.

### Phylogeny and domain structure of the *foxp3* gene in birds

The FGENESH identified *foxp3* gene in the ground tit and the corresponding loci of the peregrine and saker falcons were consistent with the *foxp3* loci of mammals, reptiles and amphibians. Failure of automated annotation pipelines to identify *foxp3* in the two falcons was attributed to the high proportion of repeat sequences, considered to represent regions of low reliability, within their genomes. The ground tit has 11 forecast *foxp3* exons; by comparison, pairwise sequence alignment of ground tit FoxP3 with the region containing the putative *foxp3* genes in the falcons by GeneWise revealed four exons in the peregrine falcon and three exons in the saker falcon (Fig 1C).

**Fig 3:**
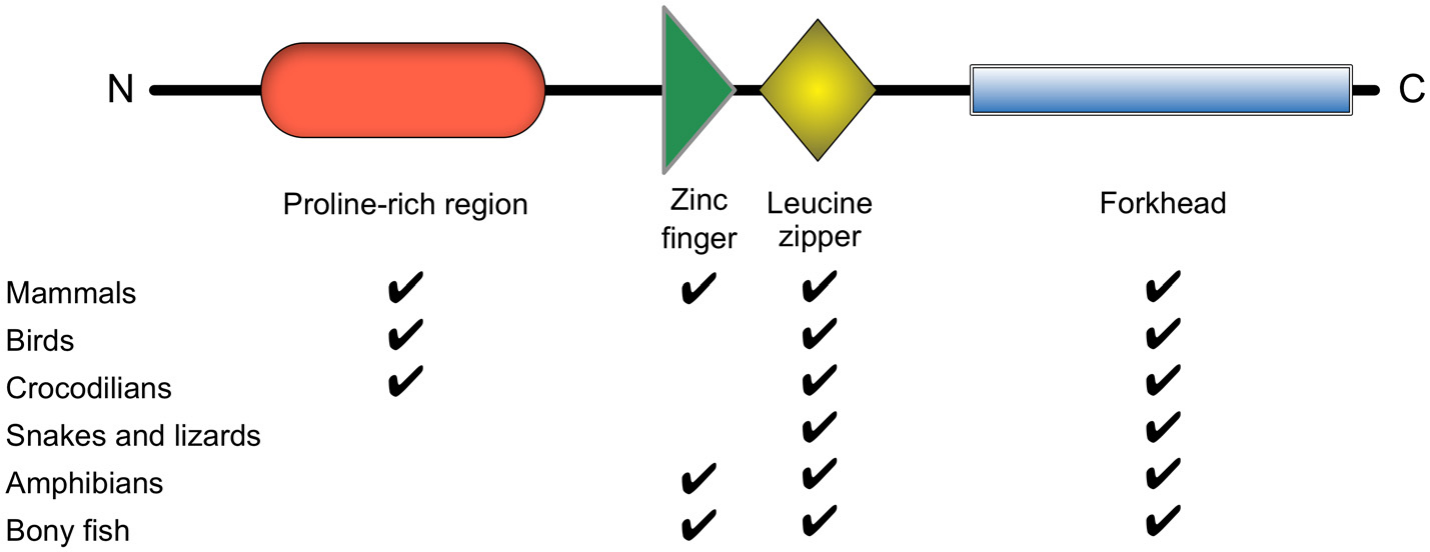
A summary of the structural domains and regions of FoxP3 in different clades of the Animal Kingdom.

There is a single predicted FoxP3 transcript in the ground tit that encodes a protein 298 amino acids in length. The highest match for this protein was to FoxP3 in the American alligator (52% sequence identity for 83% of the query sequence) using BLASTP on the NCBI nr database; the highest scoring mammalian match was to FoxP3 in the platypus (*Ornithorhynchus anatinus*; 48% sequence identity for 63% of the query sequence) and the top 100 matches were all to FoxP3 (or FoxP3-like) proteins. The region of ground tit FoxP3 extending from the middle of the protein to its C-terminus, encompassing the LZ and FRK domains, had the highest sequence identity with other FoxP3s, whereas the rest of the protein had poor sequence identity. All identified exons from both falcons mapped to a region spanning the FRK domain. Both the LZ and FRK domains are the most highly conserved regions of FoxP3 in other species, the ZF domain being conserved in mammals, fish and amphibians, but not in reptiles (Fig 3).

### The FRK domain

**Fig 4:**
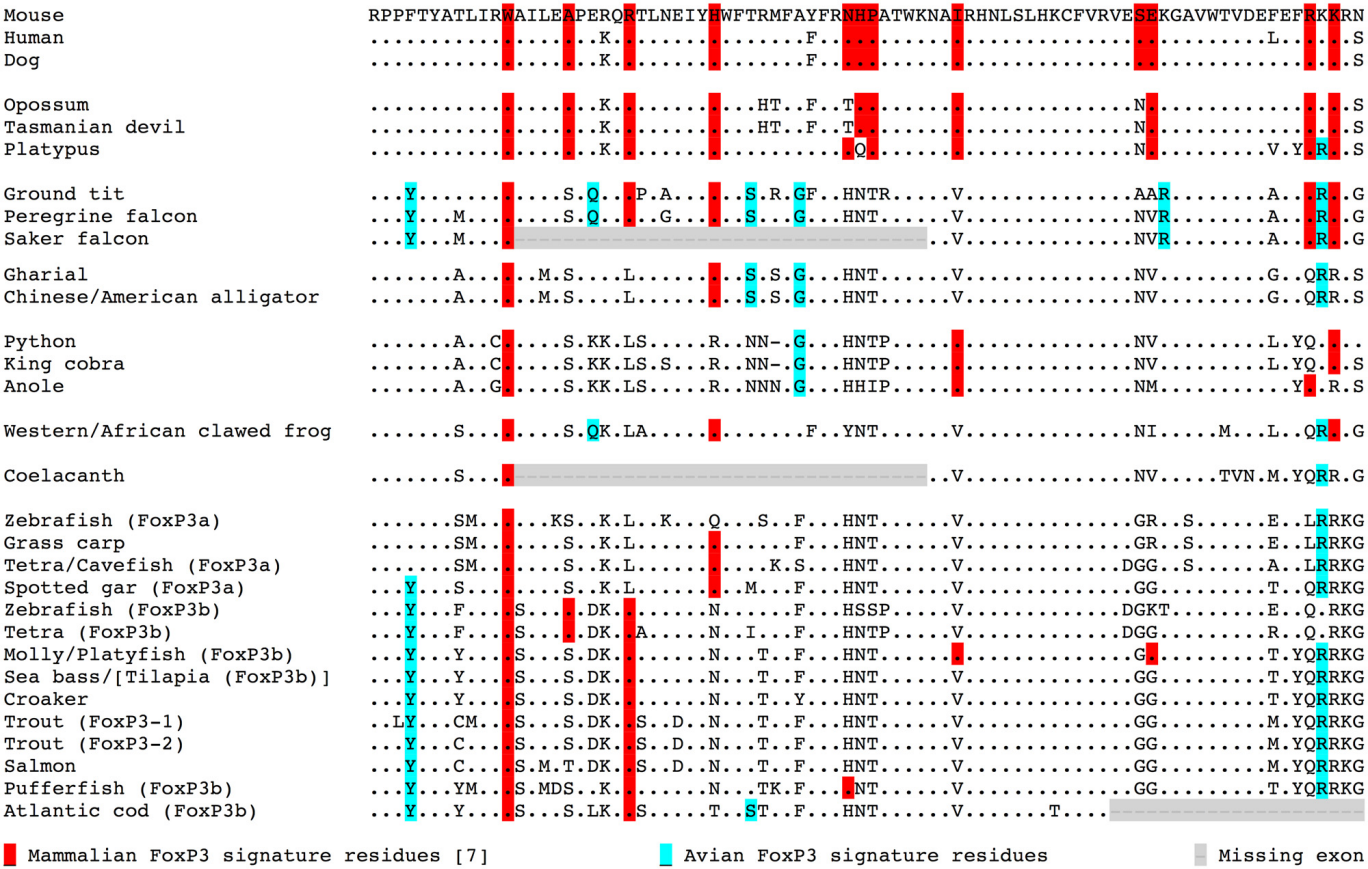
Alignment of the forkhead domain of FoxP3 and FoxP3-like proteins, highlighting mammalian and avian signature residues. The consistent exon boundaries in the ground tit and peregrine falcon were used to curate the gap within the sequence of the saker falcon. The mouse sequence is used as the baseline sequence and only amino acids in other sequences that differ are shown.

**Fig 5:**
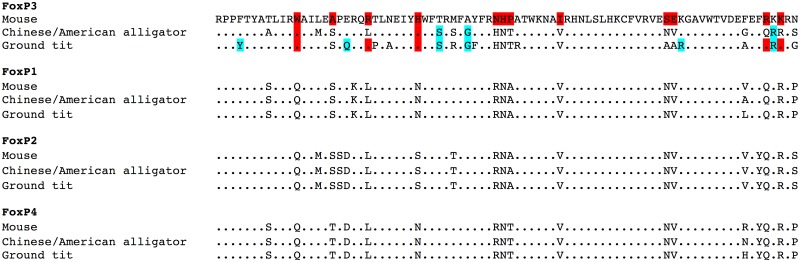
The distinct forkhead domain of FoxP3. The FoxP3 forkhead domain of the mouse, alligator and ground tit, aligned to FoxP1, FoxP2 and FoxP4 paralogues in the same species, demonstrating its divergent evolution and unique signature residues. The mouse FoxP3 sequence is used as the baseline sequence and only amino acids in other sequences that differ are shown.

A mammalian FoxP3 ‘signature’ of 12 residues has been described, incorporating nine residues that are conserved in all mammals including marsupials and monotremes [7]. Taking the complete FRK sequences of the ground tit and peregrine falcon, together with the available partial sequence of the saker falcon, we identified six conserved residues in birds that may represent a candidate avian FoxP3 ‘signature’ (Fig 4) and we also confirmed their absence in FoxP1, FoxP2 and FoxP4 FRK sequences (Fig 5). Furthermore, the birds also possess five of the 12 mammalian signature residues, which are thought to represent ‘gain of function’ mutations that play important roles in protein-protein interactions and DNA binding in mammalian FoxP3. Further work will be required to determine the robustness of the provisional avian signature across other species of birds and the functional relevance of their unique FRK residues, which may confer particular evolutionary advantages in this class of animals.

### The LZ and ZF domains

The LZ domain is present in all species having a *foxp3* gene, playing an essential role in FoxP3 homodimerization and DNA binding [52]. The ground tit and coelacanth (*Latimeria chalumnae*) have an isoleucine (I) rather than valine (V) residue at the first position of the published consensus motif V-x(6)-L-x(6)-L-x(6)-L [20].

The ZF domain was not detected in FoxP3 of the ground tit or any available reptilian species; the signal was partial, present at the C terminus of this region only, in the Western clawed frog (*Xenopus tropicalis*), attributed to a missing exon and consequent misalignment of the remaining sequence, but clearly present in the African clawed frog (*Xenopus laevis*). Of interest, the region of FoxP3 of the ground tit and three crocodilian species aligning with the ZF domain of other species showed a high proportion of proline residues, suggesting that it represented the C-terminal end of the poorly conserved ProR region. However, the quality of reptilian sequences in this syntenic region is generally poor, with sequence gaps, partial genes and missing exons. Whether the ZF domain is absent in all archosaurs, or just a subset, remains an open question, although it appears not to be essential for core functions of FoxP3 [53].

### ProR region

A ProR region is present in all mammals and its length is extended in placental mammals, but it is absent in fish and amphibians [7]. We investigated whether a ProR region is also present in birds and reptiles using all available species for which we could identify a sequence from the N-terminus to the ZF domain (or to the LZ domain in species lacking a ZF): the ground tit, the American alligator, the Chinese alligator (*A*. *sinensis*), the gharial (*Gavialis gangeticus*), the Burmese python (*Python bivittatus*), and the green anole (*Anolis carolinensis*).

The degree of proline enrichment in our dataset of avian and crocodilian species was higher than that in mammals– 25.2% in the ground tit, 20.3% in the American alligator, 21.9% in the Chinese alligator and 25.5% in the gharial. However, snakes and lizards (squamates) lacked a discernible ProR region, with proline content of only 10.8% in the green anole and 8.7% in the Burmese python, and there was minimal sequence similarity between archosaurs and mammals in this region. Birds and crocodilians may thus share a ProR region with mammals that is not present in squamates, presumably as a result of convergent evolution or to secondary loss in scaled reptiles. However, the disparity in length of the FoxP3 N-terminal region in different species was notable (from 55aa in the gharial to 267aa in the opossum), providing a note of caution to predictions of its functional adaptations between species.

### Immune regulatory pathways

Immune regulatory pathways are poorly understood in birds. Regulatory T cells have been identified in various studies by the phenotype CD4^+^CD25^+^ [25,26,54,55] and are implicated in the pathogenesis of diseases as varied as Marek’s disease, in which the neoplastic T cells have a Treg phenotype [56]; infectious bursal disease, in which there is a net migration of Tregs to the Bursa of Fabricius [57]; a number of coccidial enteropathogens, which are thought to subvert the immune response by inducing Tregs [25]; and enteric infections with *Salmonella spp*. and other food-borne zoonotic pathogens, whose persistence is thought to be promoted by the presence of Tregs [25,58]. Regulatory T cells therefore present a novel therapeutic target for a number of important diseases of poultry. A deeper understanding of the molecular determinants of Tregs in avian species will facilitate the therapeutic manipulation of these cells. As a major transcriptional factor in the transcriptomic landscape of mammalian Tregs, FoxP3 is an obvious target for small molecule, antibody and chimeric therapies of the future [59–63]. Demonstration of the impact of FoxP3 in avian Tregs would pave the way for a raft of new therapies for diseases that not only impose a significant welfare burden, but also costs the global poultry industry millions of dollars in lost revenue [64–67].

## Conclusion

Our phylogenetic analysis not only provides cogent evidence for the existence of the avian *foxp3* gene in three genomes, but also suggests that the generally low quality of avian genomes in the *foxp3* region has contributed to the failure of previous studies to detect the gene in Aves. Further, the partial *foxp3* transcriptomic data for the ground tit provide evidence of *foxp3* expression in at least one bird. This represents a significant step forward in our understanding of a pivotal immune regulatory pathway in birds. It also highlights the evolutionary conservation in archosaurs and squamates of regions of known functional importance, including the residues of the FRK, suggesting that they are subject to on-going or recent purifying selection. Moreover, there is also RNA-Seq data for the Chinese and American alligators, green anole and Burmese python demonstrating that all the annotated genomic features of *foxp3* are expressed (XM_006031993.1, XM_006261277.1, XM_008103925.1 and XM_007420528.1).

Further work is required to confirm that FoxP3 is present in all avian species and to determine whether a distinct set of conserved residues is present in all, or phylogenetic subsets, of these species, as well as to verify its regulatory function.

## Supporting Information

S1 File Ground tit *foxp3* FGENESH gene prediction. (PDF)

S2 File Ground tit muscle *foxp3* SRA reads from SRX246872. (TXT)

S1 Table Mammalian, reptilian and amphibian NCBI genome information. (XLSX)

S2 Table Avian NCBI genome information. (XLSX)

S3 Table FoxP3 proline enrichment in birds, bony fish and reptiles. (XLSX)
