# Supplementary material for: Missed, Not Missing: Phylogenomic Evidence for the Existence of Avian FoxP3
Source: PLoS One. 2016 Mar 3;11(3):e0150988. doi: 10.1371/journal.pone.0150988 (PMC4777427; doi:10.1371/journal.pone.0150988)
Supplement: S1 File — (PDF) [file pone.0150988.s001.pdf]

FGENESH 2.6 Prediction of potential genes in Falco_cherrug genomic DNA
Seq name: gi|539359005:94192-99928 Pseudopodoces humilis unplaced genomic scaffold, PseH
Length of sequence: 5737
Number of predicted genes 1: in +chain 1, in -chain 0.
Number of predicted exons 11: in +chain 11, in -chain 0.
Positions of predicted genes and exons: Variant 1 from 1, Score:102.471936

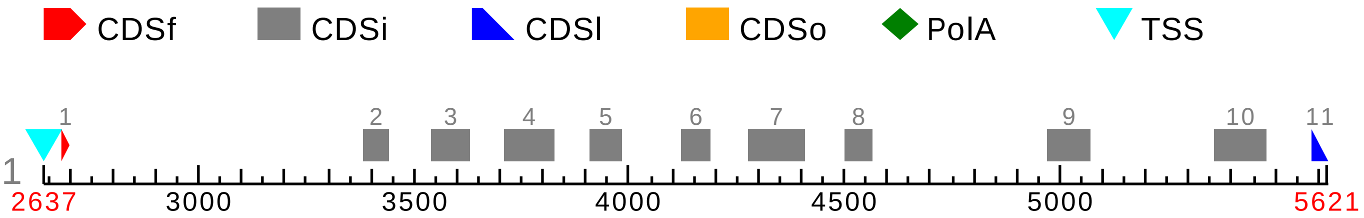

1	+		TSS	2637			1.25				
1	+	1	CDSf	2678	-	2697	-1.11	2678	-	2695	18
1	+	2	CDSi	3379	-	3439	7.05	3380	-	3439	60
1	+	3	CDSi	3537	-	3627	2.33	3537	-	3626	90
1	+	4	CDSi	3706	-	3823	3.45	3708	-	3821	114
1	+	5	CDSi	3905	-	3980	14.09	3906	-	3980	75
1	+	6	CDSi	4118	-	4186	17.74	4118	-	4186	69
1	+	7	CDSi	4273	-	4405	7.73	4273	-	4404	132
1	+	8	CDSi	4498	-	4562	15.72	4500	-	4562	63
1	+	9	CDSi	4968	-	5069	8.00	4968	-	5069	102
1	+	10	CDSi	5356	-	5477	33.18	5356	-	5475	120
1	+	11	CDSl	5582	-	5621	-0.43	5583	-	5621	39

Predicted protein(s):

```
>FGENESH:[mRNA] 1 11 exon (s) 2678 - 5621 897 bp, chain +
ATGGCGGGCACCCGGGACCCCCCCAGGAGCGGCCCACGGTGCTGACCCGCGCGGACGGG
GGGGTCCCGCGGCGCTCGCAGGTGCCCCCCGAGGGGGGGTCGTGCCCGCCCCTCCTGCAC
GTGCAGCACCCCGGGGTGTTGGTGATGCGGACCCGGCTGCCCCCCGCGCACGGTCTCCCC
GCTTTTGAATGGGGCCCCAAAGACCCCCCCGGCCGCGACAGAGACCCCTCCCCCGCCCCT
GCCGGCAGCGACCCCCCCCCGCCGCCCCCCGGCGCTGCGCAGCCCCCCAGGCCCCCCCGC
CGTGAGCCGGGGGAGGTGGCTCAGTGCCTGCGGCAGCTGGAGAGGATCCAGGAGCTGGAG
CAGCAGCTGGCCCGGGAGCGGCACCGCCTGGGGCTGTTACAGGCGCAGCTCCTCCGGAGG
AGCCCCCCCGGCACGGGCCCCCCCGGGAAGGGCCAGGCTGGGCTCCCCCCGATTTGGGGT
CCTGCAGCCGCCCCCGAGGCCGAGGGGGACCCCGAGATGATGCTGCCCCCCCCGGGGCAC
CCCTGGGAGCGTGGGGGGCTCTACCCAGAACTGGAGTATTACCGGCTCAGCACCGTGCGG
CCCCCCTACACCTACGCCACGCTCATCCGCTGGGCCATCCTGGAGTCCCCGCAGCGGCAG
CGCCCCCTGGCGGAGATTTACCACTGGTTCAGCCGCAGGTTCGGCTTCTTCCGGCACAAC
ACCCGCACCTGGAAGAACGCCGTGCGCCACAACCTGAGCCTGCACAAGTGCTTCGTGCGG
GTGGAGGCCGCTCGCGGCGCCGTCTGGACCGTGGACGAGGCCGAGTTCCGGCGCAAGCGG
GGCCAGCACTACCCCAGGGACTGTGACCTCAAGTACTTTATGCCCCCCCGGAGCTGA
>FGENESH: 1 11 exon (s) 2678 - 5621 298 aa, chain +
MAGTRDPPQERPTVLTRADGGVPRRSQVPPEGGSCPPLLHVQHPGVLVMRTRLPPAHGLP
AFEWGPKDPPGRDRDPSPAPAGSDPPPPPPGAAQPPRPPRREPGEVAQCLRQLERIQELE
QQLARERHRLGLLQAQLLRRSPPGTGPPGKGQAGLPPIWGPAAAPEAEGDPEMMLPPPGH
PWERGGLYPELEYYRLSTVRPPYTYATLIRWAILESPQRQRPLAEIYHWFSRRFGFFRHN
```

TRTWKNAVRHNLSLHKCFVRVEAARGAVWTVDEAEFRRKRGQHYPRDCDLKYFMPPRS
